# Cysteamine with In Vitro Antiviral Activity and Immunomodulatory Effects Has the Potential to Be a Repurposing Drug Candidate for COVID-19 Therapy

**DOI:** 10.3390/cells11010052

**Published:** 2021-12-24

**Authors:** Tonino Alonzi, Alessandra Aiello, Linda Petrone, Saeid Najafi Fard, Manuela D’Eletto, Laura Falasca, Roberta Nardacci, Federica Rossin, Giovanni Delogu, Concetta Castilletti, Maria Rosaria Capobianchi, Giuseppe Ippolito, Mauro Piacentini, Delia Goletti

**Affiliations:** 1 Translational Research Unit, National Institute for Infectious Diseases “Lazzaro Spallanzani”-IRCCS, 00149 Rome, Italy; tonino.alonzi@inmi.it (T.A.); alessandra.aiello@inmi.it (A.A.); linda.petrone@inmi.it (L.P.); saeid.najafi@inmi.it (S.N.F.); delia.goletti@inmi.it (D.G.); 2 Department of Biology, University of Rome “Tor Vergata”, 00133 Rome, Italy; manuela.deletto@gmail.com (M.D.); federica.rossin@uniroma2.it (F.R.); 3 Laboratory of Electron Microscopy, National Institute for Infectious Disease “Lazzaro Spallanzani”-IRCCS, 00149 Rome, Italy; laura.falasca@inmi.it (L.F.); roberta.nardacci@inmi.it (R.N.); 4 Institute of Microbiology, Università Cattolica del Sacro Cuore, 00168 Rome, Italy; giovanni.delogu@unicatt.it; 5 Mater Olbia Hospital, 07026 Olbia, Italy; 6 Virology Unit, National Institute for Infectious Disease “Lazzaro Spallanzani”-IRCCS, 00149 Rome, Italy; concetta.castilletti@inmi.it (C.C.); maria.capobianchi@inmi.it (M.R.C.); 7 Scientific Direction, National Institute for Infectious Disease “Lazzaro Spallanzani”-IRCCS, 00149 Rome, Italy; giuseppe.ippolito@inmi.it

**Keywords:** SARS-CoV-2, COVID-19, cysteamine, cystamine, antiviral, drug repurposing

## Abstract

The ongoing pandemic of coronavirus disease-2019 (COVID-19), caused by severe acute respiratory syndrome coronavirus 2 (SARS-CoV-2), needs better treatment options both at antiviral and anti-inflammatory levels. It has been demonstrated that the aminothiol cysteamine, an already human applied drug, and its disulfide product of oxidation, cystamine, have anti-infective properties targeting viruses, bacteria, and parasites. To determine whether these compounds exert antiviral effects against SARS-CoV-2, we used different in vitro viral infected cell-based assays. Moreover, since cysteamine has also immune-modulatory activity, we investigated its ability to modulate SARS-CoV-2-specific immune response in vitro in blood samples from COVID-19 patients. We found that cysteamine and cystamine decreased SARS-CoV-2-induced cytopathic effects (CPE) in Vero E6 cells. Interestingly, the antiviral action was independent of the treatment time respect to SARS-CoV-2 infection. Moreover, cysteamine and cystamine significantly decreased viral production in Vero E6 and Calu-3 cells. Finally, cysteamine and cystamine have an anti-inflammatory effect, as they significantly decrease the SARS-CoV-2 specific IFN-γ production in vitro in blood samples from COVID-19 patients. Overall, our findings suggest that cysteamine and cystamine exert direct antiviral actions against SARS-CoV-2 and have in vitro immunomodulatory effects, thus providing a rational to test these compounds as a novel therapy for COVID-19.

## 1. Introduction

Coronavirus disease 2019 (COVID-19), which is caused by the novel coronavirus severe acute respiratory syndrome coronavirus 2 (SARS-CoV-2), is a global concern due to its high transmissibility and pathogenicity [1]. While great efforts have been focused on vaccine development, many unanswered questions remain regarding the development of an effective therapy [2]. The repurposing of drugs demonstrated to be safe, effective, and approved in humans for other indications, has been proposed as a strategy to accelerate the identification of compounds that can cure or prevent COVID-19 [3]. Thiol-containing compounds display potent biological effects, such as anti-oxidant, anti-inflammation, and protection from bacterial infections [4,5,6,7]. Cysteamine, also known as 2-mercaptoethylamine or aminoethanethiol, is an aminothiol endogenously synthesized by human cells through the cleavage of pantetheine to form cysteamine and pantothenate during coenzyme A metabolism [8].

Cysteamine and its disulfide product of oxidation, cystamine, exert many biological effects; for example, they can act as exogenous supplements for L-cysteine transport into the cells for the synthesis of glutathione, an important antioxidant agent. These compounds, therefore, influence the oxidative state of the cells, regulating several signaling pathways involved in the important mechanisms of cellular homeostasis [9]. Moreover, it has been reported that cysteamine targets many cellular enzymes for its ability to modify disulfide bonds or susceptible cysteine residues. Among those, cysteamine has been shown as one of the earliest known pharmacological inhibitors of transglutaminase 2 (TG2) [10], an ubiquitous enzyme involved in several important cellular processes, such as cell death/survival and autophagy [6].

Cysteamine has been approved by the Food and Drug Administration (FDA) and the European Medicines Agency (EMA) for the treatment of nephropathic cystinosis, a rare inherited autosomal recessive disease caused by mutations in the lysosomal cystine carrier cystinosin, which lead to intralysosomal cystine accumulation. Cysteamine converts cystine into both cysteine and cysteamine–cysteine mixed disulfide, which are released from lysosomes via the lysine and cysteine transport system [11]. More recently, cysteamine has been tested in several clinical trials and a good safety profile has been shown for the treatment of several diseases, such as inherited mitochondrial disease, cystic fibrosis (CF), neurodegenerative disorders, major depressive disorder, asthma, non-alcoholic fatty liver disease (NAFLD) and inflammatory bowel disease (https://clinicaltrials.gov/ct2/results?term=cysteamine&Search=Search, Last accessed on 22 December 2021).

In addition to these therapeutic indications, cysteamine has anti-infective properties. It increases *Pseudomonas aeruginosa* sensitivity to antibiotics exerting activity against different Plasmodium species, and has mucolytic-antimicrobial activity in cystic fibrosis [12]. Recently, we showed that the pharmacological inactivation of TG2 by cysteamine enhances the anti-mycobacterial properties of *Mycobacterium tuberculosis* (Mtb)-infected macrophages acting as a host-directed agent [13,14]. Cysteamine exhibited an improved clearance of, and resistance to, *Pseudomonas aeruginosa* in both patients and mice with CF [5]. Cysteamine has also immune-modulatory activity, as shown in asthma and CF [15,16,17].

Interestingly, cysteamine and cystamine show the ability to inhibit the in vitro replication of human immunodeficiency virus type 1 (HIV) [18,19], influenza A virus H1N1 [20] and, very recently, of SARS-CoV-2 [21]. However, no conclusive evidence of the mechanisms of the anti-SARS-CoV-2 activity were provided.

Based on this evidence of the antiviral and immune modulating effects, the aim of this study was to characterize the impact of cysteamine and cystamine against SARS-CoV-2. Moreover, we investigated their ability to modulate the SARS-CoV-2-specific immune response in vitro in COVID-19 patients, using a whole-blood assay platform [22,23,24].

## 2. Materials and Methods

### 2.1. Study Population

The study was approved by the Ethical Committee of the National Institute for Infectious Diseases (INMI) “Lazzaro Spallanzani”-IRCCS (approval number 59/2020), and conducted between 14 April and 24 May. The inclusion criteria for the enrollment of COVID-19 patients were a diagnosis based on a positive RT-PCR from a nasopharyngeal swab for SARS-CoV-2 and a disease with the clinical characteristics already described [25]. The exclusion criteria were: HIV infection, inability to sign an informed consent, and <18 years of age. All patients provided informed written consent. Demographic and clinical information were collected at enrollment.

### 2.2. Cells, Drugs, and Stimuli

Vero E6 cells, the kidney epithelial cell line originally established from an African green monkey (*Chlorocebus* sp.; formerly called *Cercopithecus aethiops*), were cultured in Minimum Essential Medium (MEM) supplemented with heat-inactivated 10% fetal bovine serum (FBS), 2 mM L-glutamine, and 1% penicillin/streptomycin solution (Merck Life Science, Milan, Italy, Cat. No. M2279; F7524; G7513; P0781, respectively) and maintained at 37 °C in 5% CO_2_. The human epithelial lung adenocarcinoma Calu-3 cells were cultured in RPMI 1640 (Merck Life Science, Cat. No. R0883) supplemented with heat-inactivated 10% FBS, 2 mM L-glutamine, and 1% penicillin/streptomycin solution. All cell lines were maintained in 5% CO_2_ at 37 °C.

Generation of the TG2-deficient Huh7 cell line (Huh7-TG2 KO) and its control (Huh-7-CTR) was obtained by the CRISPR-Cas9 system. CRISPR-CAS9 lentiviral vectors specific for either TG2 (TGM2 sgRNA, ABMGood, Richmond, BC, Canada, Cat. No. K2366205) or control (Scramble sgRNA, ABMGood Cat. No. K010) were produced in HEK293T cells by co-transfecting 10 μg of lentiviral vectors with 2.5 μg of pVSV-G plasmids and 7.5 μg of psPAX2 plasmids, using the calcium phosphate method. After 48 h, lentiviral particles were harvested, filtered through a 0.45 μm membrane, and used to transduce Huh7 cells. For stable clones, Huh7 cells were selected with puromycin (2 μg/mL) and a single clone was picked out.

Cysteamine (CAS 60-23-1; Merck Life Science Cat. M9768), cystamine (CAS 56-17-7; Merck Life Science Cat.30050) were dissolved in H_2_O, filtered through a 0.22 µm membrane, and used at the indicated concentrations by dilution in medium. Z-DON (Z-DON-Val-Pro-Leu-OMe. Zedira GmbH Darmstadt, Germany; Cat. Z006) was dissolved in 100% DMSO and diluted in medium.

To evaluate the SARS-CoV-2 specific response, commercial peptide pools (all from Miltenyi Biotec, Bergisch Gladbach, Germany) constituted by 15-mer peptides with 11 amino acid overlap covering the sequence of the SARS-CoV-2 isolate Wuhan-Hu-1 (GenBank MN908947.3) were used: pool S (spike protein (QHD43416.1), including PepTivator^®^ SARS-CoV-2 Prot_S1, Prot_S, and Prot_S+; Cat. 130-127-048, Cat. 130-126-701, and Cat. 130-127-312, respectively); pool N (nucleocapsid phosphoprotein (QHD43423.2), PepTivator^®^ SARS-CoV-2 Cat. 130-126-699); and pool M (membrane glycoprotein (QHD43419.1), PepTivator^®^ SARS-CoV-2 Prot_M. Cat. 130-126-703) [26]. Staphylococcal enterotoxin B (SEB) (Merck Life Science Cat. S4881) was used as positive control.

### 2.3. Whole-Blood Assay

Whole-blood assay was performed as we recently described [22,23,26]. Briefly, whole blood (600 µL) was stimulated for 20–24 h in a 48-well flat-bottom plate at 37 °C (5% CO_2_) with or without pool S (0.1 µg/mL) or pool N (1 µg/mL) or pool M (0.1 µg/mL) or SEB antigen (200 ng/mL). Cysteamine 400 µM/mL or cystamine 200 µM/mL was added concomitantly with the antigen stimulation. After stimulation, plasma was harvested and frozen at −80 °C until IFN-γ evaluation. IFN-γ was measured by an automatic ELISA system (ELLA, R&D System, Minneapolis, MN, USA, Cat. SPCKB-PS-002574). The detection limit of this assay was 0.17 pg/mL. IFN-γ values were subtracted from the unstimulated control.

### 2.4. Virus Protection Assay

The antiviral activity of cysteamine, cystamine, or Z-DON was tested by cytopathic effects (CPE) inhibition assay using Vero E6 cells. The determination of the in vitro susceptibility of SARS-CoV-2 to those compounds is expressed as percentage of surviving cells, as we recently described [27]. Briefly, Vero E6 cells were washed with 1× PBS, dislodged with trypsin-EDTA solution (Merck Life Science, Cat. No. T3924), and seeded into 96-well imaging plates at a density of 2.5 × 10^4^ cells per well in culture medium, as indicated above. The cells were incubated for 24 h to allow adherence. Cells were treated 1 h before SARS-CoV-2 infection with either a 1:2 serial dilution of cysteamine, cystamine, or H_2_O as control or with 1:3 serial dilution of Z-DON or DMSO as control. Cells were infected at 0.001 multiplicity of infection (MOI; which reflects the ratio of PFU to the number of infected cells) using MEM supplemented with heat-inactivated 2% FBS and 2 mM L-glutamine in the presence of the different treatments. After 1 h incubation, the viral input was replaced by fresh medium containing the different compounds/controls. Cells were then treated every 24 h by adding the drugs to the culture medium, incubated at 37 °C with 5% CO_2_ for 72 h, and cell viability was evaluated by a standard crystal violet staining assay and measuring the optical density (OD) at 595 nm. Results were analyzed using GraphPad Prism 7.04 (GraphPad, San Diego, CA, USA) and reported as the percentage of surviving cells compared to the uninfected cells.

In some experiments, cells were treated using different protocols in terms of time of drug administration in respect to SARS-CoV-2 infection. In particular, Vero E6 cells were: (i) pre-treated for 1 h and then infected for 1 h in the absence of drugs (MOI = 0.001) for pre-treatment protocol; (ii) infected for 1 h in the presence of drugs (MOI = 0.001) for co-treatment protocol; and (iii) infected for 1 h in the absence of drugs (MOI = 0.001), virus removed and drugs added at 1 h.p.i. for post-treatment protocol. Afterwards, the virus was removed and the cells were cultured in the presence of compounds that were added 1 h, 24 h, and 48 h after SARS-CoV-2 infection, as described above, until the end of the experiments (72 h.p.i.).

### 2.5. SARS-CoV-2 Virus Yield Assay

The ability of the test compounds to reduce the SARS-CoV-2 replication was explored by a typical virus yield reduction assay. The virus strain isolated at INMI L. Spallanzani IRCCS (2019-nCoV/Italy-INMI1; GenBank MT066156) [28] was used in all the experiments performed with the different cell lines (Vero E6, Calu-3, or Huh7). Virus replication in the various experimental conditions was measured by back-titrating the culture supernatants of the infected cells by limiting dilution assay in Vero E6 cells, as we recently described [27]. Readout of the virus back-titration was based on detection of CPE, and infectious titer was expressed as 50% tissue-culture effective dose (TCID50) values, calculated according to the Reed–Muench method.

### 2.6. Viral RNA Quantification

The viral RNA present in the culture medium of the samples described above were quantified by Ct measurement with Simplexa™ COVID-19 Direct assay (DiaSorin, Vicenza, Italy), as previously described [29] according to the manufacturer’s instructions. Briefly, 50 μL of culture medium and 50 μL of Reaction Mix were added to their specific wells on a direct amplification disk, which was loaded onto the LIAISON^®^ MDX instrument (DiaSorin).

### 2.7. Transmission Electron Microscopy

Transmission electron microscopy (TEM) was performed on cultured cells using standard procedures. Cultured cells were fixed with 2.5% glutaraldehyde in 0.1 M cacodylate buffer, for 4 h at 4 °C. Post-fixation was performed with 1% OsO4. Samples were then dehydrated in graded ethanol and embedded in Epon resin, as previously described [30,31]. Ultrathin sections were stained with 2% uranyl acetate and observed under a transmission electron microscope (JEOL JEM 2100 Plus, Japan Electron Optics Laboratory Co. Ltd., Tokyo, Japan). Images were captured digitally with a digital camera TVIPS (Tietz Video and Image Processing Systems GmbH, Gauting, Germany). Quantitative analysis of infected cells was assessed in blind by two authors counting 100 cells per condition.

### 2.8. Immunoblotting Analysis

Western blotting analysis was carried out as previously reported [32]. Whole cell extracts (10 μg), obtained using CelLytic™ MT Cell Lysis Reagent (Merck Life Science, Cat.No. C3228), were separated on SDS-PAGE 10% gels and electroblotted onto nitrocellulose membranes (Merck Life Science, Cat.No. GE10600041). Blots were incubated with primary antibodies in 5% nonfat dry milk (Biosigma, Venezia, Italy, Cat.No. 711160) in PBS (Thermo Fisher Scientific, Waltham, MA, USA, Cat.No. J61196.AP) plus 0.1% Tween-20 (Merck Life Science, Cat.No. P1379) overnight at 4 °C. The primary antibodies used in this study were anti-TG2 (Thermo Fisher Scientific Cat.No. MA5-12739) and anti-actin (Merck Life Science, Cat.No. A-2066). Detection was achieved using horseradish peroxidase-conjugated secondary antibodies (anti-mouse 715-036-150 or anti-rabbit 711-036-152; Jackson ImmunoResearch Laboratories, Ely, UK) and enhanced chemiluminesence (ECL) [Immobilon Classico Cat.No. WBLUC0500 and Immobilon Crescendo Western HRP substrate Cat.No. WBLUR0500 Merck Life Science]. Signals were acquired using a ChemiDoc imaging system (Bio-Rad, Milan, Italy).

### 2.9. Statistical Analysis

Data were analyzed using GraphPad Prism 7.04 (GraphPad) using Wilcoxon matched-pairs rank test. Medians and interquartile ranges (IQR) were calculated for continuous measures; the Friedman test was used for comparisons among groups and the Wilcoxon matched-pairs rank test with Bonferroni correction for pairwise comparisons.

## 3. Results

### 3.1. Cysteamine and Cystamine Significantly Reduce the Cytopathic Effect Induced by SARS-CoV-2 in Vero E6 Cells

We characterized the impact of cysteamine and cystamine on SARS-CoV-2 by evaluating their effect on the virus-induced cytopathic effect (CPE) in Vero E6 cells. Cells were treated with either cysteamine or cystamine using two-fold serial dilutions of compounds ranging from 2000 μM to 31.25 μM, or with the vehicle [H_2_O from 2% to 0.031% (*v*/*v*)] as the control.

**Figure 1:**
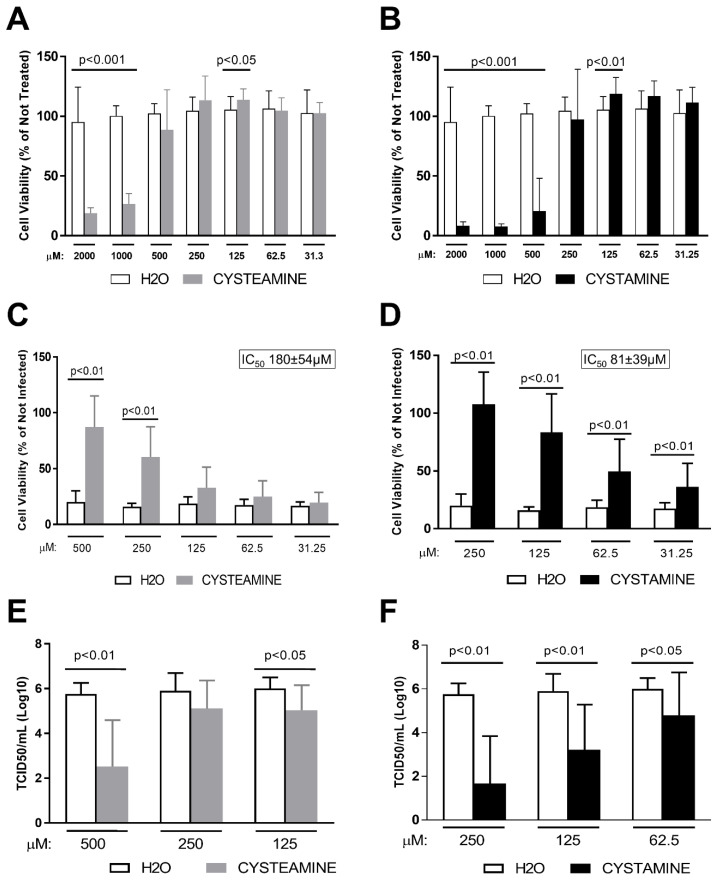
Cysteamine and cystamine inhibited SARS-CoV-2-induced CPE and decreased viral production in Vero E6 cells. (**A**–**D**) Vero E6 cells were treated with different doses of cysteamine (panels **A**,**C**) and cystamine (panels **B**,**D**) (from 2000 to 31.25 μM; 1:2 serial dilutions) or H_2_O as control (from 2 to 0.031% *v*/*v*) 1 h before SARS-CoV-2 infection. Absorption of the virus was allowed for 1 h at 37 °C in presence of the different treatments. The unabsorbed virus was removed and replaced by fresh medium with cysteamine, cystamine or H_2_O as above. Cells were then treated every 24 h and incubated at 37 °C with 5% CO_2_ for 72 h when the survival of not infected (**A**,**B**) or infected (**C**,**D**) cells was measured by crystal violet staining assay. The results were evaluated setting the not infected cells as 100% and the remaining values represented as a relative value. Experiments were performed in triplicate and data are expressed as mean ± S.D. (n = 6). In (**C**,**D**) 50% inhibitory concentration (IC_50_) values are reported. (**E**,**F**) Culture medium from SARS-CoV-2-infected Vero E6 cells, treated as indicated, were collected at 72 h.p.i. Virus yield was measured by back-titration of supernatants as described in the methods. Virus titers are expressed as 50% tissue-culture effective dose (TCID_50_/mL) values according to the Reed–Muench method. Statistically significant differences between compound- and H_2_O-treated cells have been calculated using Wilcoxon matched-pairs rank tests.

Firstly, we assessed the safety of cysteamine or cystamine treatment of Vero E6 cells, and found that high concentrations of both compounds had toxic effects, with a 50% cytotoxic concentration (CC_50_) of 660 ± 243 μM (Figure 1A) and 357 ± 68 μM, respectively (Figure 1B). Therefore, for the CPE assay we considered only the non-toxic concentrations with ranges of 500–31.25 μM for cysteamine and 250–31.25 μM for cystamine. As shown in Figure 1C and D, both compounds significantly prevented the SARS-CoV-2-induced CPE in a dose-dependent manner. In particular, cysteamine significantly reduced the CPE at 500 μM and 250 μM compared to drug-vehicle (H_2_O) (Figure 1C), with a 50% inhibitory concentration (IC_50_) value of 180 ± 54 μM. On the other hand, cystamine significantly reduced the CPE from 250 μM to 31.25 μM with an IC_50_ value of 81 ± 39 μM (Figure 1D).

### 3.2. Cysteamine and Cystamine Significantly Reduce SARS-CoV-2 Production in Vero E6 Cells

We evaluated whether these compounds directly impact the SARS-Cov-2 life cycle, measuring the amount of the infectious viral particles released in the culture medium at 72 h post-infection of cells treated with cysteamine (500 μM, 250 μM and 125 μM), cystamine (250 μM, 125 μM and 62.5 μM) or with the drug-vehicle (H_2_O). Notably, cysteamine significantly reduced the yield of infectious SARS-CoV-2 mainly when used at 500 μM (Figure 1E), while cystamine significantly reduced viral yield at all the concentrations tested (Figure 1F).

We also quantified viral RNA present in the culture medium by qRT-PCR. As shown in Supplementary Figure S1, a significant reduction of viral RNA was found only at the higher concentration tested of either cysteamine or cystamine (panels A and B, respectively), thus indicating that non-infectious RNA is present in culture supernatants. We also measured viral RNA by time-course experiments treating cells with the maximum tolerated concentration of cysteamine or cystamine (500 µM or 250 µM, respectively). The inhibitory effect of viral production mediated by the drugs was already evident at 24 h.p.i. and increased by the time of culture (Supplementary Figure S1C,D).

**Figure 2:**
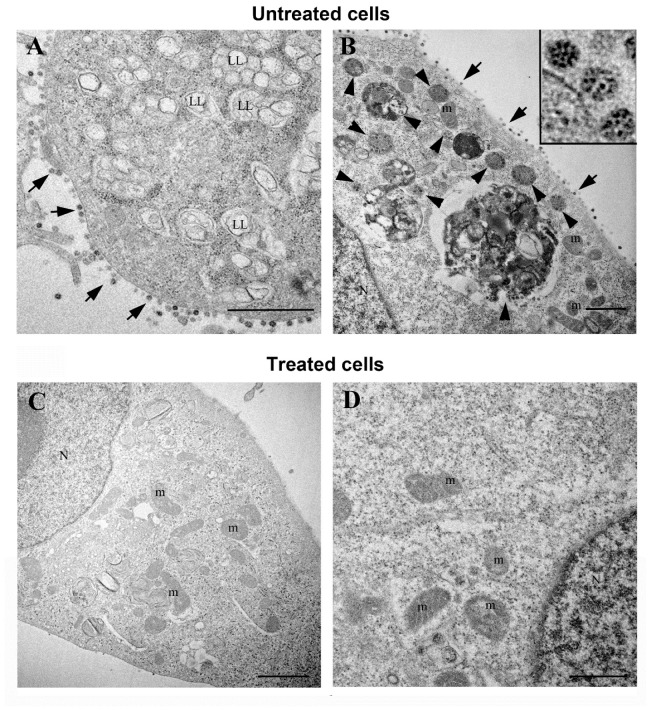
Electron microscopy images of SARS-CoV-2-infected Vero E6 cells. (**A**) Vero E6 cells at 48 h post-infection showed numerous viruses associated to the plasma membrane (arrows). A great number of vacuoles are observed in the cytoplasm, many of them found in lipolysomes (LL). Numerous ribosomes are grouped in the cell cytosol. (**B**) SARS-CoV-2 particles were also found enclosed in membrane bound vacuoles with different size and shape (arrowheads). Viruses were visible along the plasma membrane (arrows). Higher magnification of SARS-CoV-2 particles showed the characteristic black dots, corresponding to the helical nucleocapsid within the envelope (inset panel). (**C**,**D**) Vero E6 cells at 48 h post-infection treated with cysteamine (500 µM) showed a regular morphology and well preserved cytoplasmic organelles. These cells did not display signs of virus presence. N, nucleus; Nu, nucleolus; m, mitochondria; LL, lipolysosomes. Scale bars: (**A**,**B**) = 1 µm; (**C**) = 2 µm; **D** = 500 nm.

We also performed a transmission electron microscopy analysis of Vero E6 cells at 48 h.p.i., before the virus-mediated CPE was evident. Without treatment, 100% of cells showed a great number of viral particles, whereas drug treatment reduced it up to only 34% (Supplementary Figure S2B). In the untreated infected cells, we observed the presence of large amounts of viral particles along the cell surface (Figure 2A,B). Virus particles showed the characteristic black dots, due to cross-section through the viral nucleocapsid, while spikes were not always visible (Figure 2B inset). Infected cells became round in shape and displayed several intracellular changes. Ribosomes appear often grouped rather than randomly diffuse in the cytoplasm, and a higher number of vacuoles are observed, many of them consisting of lipolysomes (Figure 2A). SARS-CoV-2 particles were enclosed in membrane-bound vacuoles with different size and shape. Some of these vacuoles also contained membrane wraps and electron dense lipid materials (Figure 2B). On the other hand, the majority of infected cells treated with cysteamine (500 µM) displayed a regular morphology, with normal elongated shape and well preserved cytoplasmic organelles with no signs of virus presence (Figure 2C,D). In cysteamine-treated cells we found rare viral particles and a lower number of intracytoplasmic vacuoles containing virions (Supplementary Figure S2A). Importantly, in those cells we found the characteristic change associated to virus presence such as the lipolysosomes. Cells not infected and treated with cysteamine alone did not show signs of ultrastructural damage and were used as internal controls (Supplementary Figure S3).

Overall, cysteamine and cystamine significantly decreased SARS-CoV-2 replication, as shown by a decrease of the viral-induced CPE and by the analysis of the electron microscopy images.

### 3.3. Cysteamine and Cystamine Significantly Reduce SARS-CoV-2-Induced CPE in Vero E6 Cells, Independent of the Time of Treatment

It has been shown that cysteamine and several other thiol-based compounds decrease the binding of the spike protein to ACE2 receptor [21]. Therefore, here, we evaluated whether the inhibition of virus replication by cysteamine or cystamine was exerted by inhibiting the viral entry into Vero E6 cells. To this aim, different protocols of drug treatments were used: (i) 1 h before infection (pre-treatment); (ii) concomitantly with the infection (co-treatment); and (iii) 1 h after infection (post-treatment).

**Figure 3:**
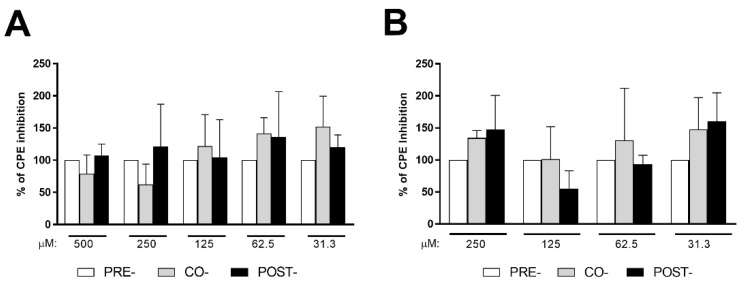
Cysteamine and cystamine inhibited SARS-CoV-2-induced CPE in Vero E6 cells independent of the time of treatment. (**A**,**B**) Vero E6 cells were treated with different doses of cysteamine (panel **A**) or cystamine (panel **B**), as indicated, in respect to SARS-CoV-2 infection (MOI = 0.001): (i) 1 h before infection (pre-treatment; white columns), (ii) during infection (co-treatment; grey columns) and (iii) 1 h after infection (post-treatment; black columns), as described in the text. Cells were then treated every 24 h and incubated at 37 °C with 5% CO_2_ for 72 h when the survival of infected cells was measured by crystal violet staining assay. The results were evaluated by setting the 1 h pre-treated cells as the 100% of CPE-inhibition activity of either cysteamine or cystamine.

As shown in Figure 3, no significant differences in the extent of the CPE inhibition were found between the pre-treatment regimen, used as a reference (100% of CPE inhibition activity), and either the co-treatment or post-treatment protocols when cysteamine (Figure 3A) or cystamine (Figure 3B) were used. Overall, our results suggest that these compounds exert their antiviral activity through cellular-mediated mechanisms rather than inhibiting viral entry.

Since cysteamine is commonly used as a TG2 inhibitor [10,13,14], we investigated whether TG2 could play a role on SARS-CoV-2 replication. Firstly, we used Z-DON, a specific inhibitor of the transamidating activity of TG2 [13], in the CPE inhibition assay in Vero E6 cells. Interestingly, Z-DON did not show any inhibition of the SARS-CoV-2-induced CPE (Supplementary Figure S4B). We also analyzed the viral production of Huh7 cells, a hepatoma cell line known to support SARS-CoV-2 replication [33], in which the TG2 genes have been deleted with CRISPR/Cas9 technology (Huh7-TG2-KO). Since SARS-CoV-2 did not induce CPE on this cell line, we evaluated the viral production measuring the infectious virus yield (TCID_50_), as described above using Vero E6 cells. Huh7-TG2-KO cells and their control (Huh7-CTR), were infected with SARS-CoV-2 (MOI = 1 and MOI = 0.1) for 48 h, when the culture medium was collected and used for back-titration of virus yield. No significant differences were found in the viral production of Huh7-TG2-KO and Huh7-CTR cells (Supplementary Figure S4C), thus demonstrating that TG2 does not have any effect on SARS-CoV-2 replication.

### 3.4. Cysteamine and Cystamine Significantly Reduce SARS-CoV-2 Production in Calu-3 Cells

**Figure 4:**
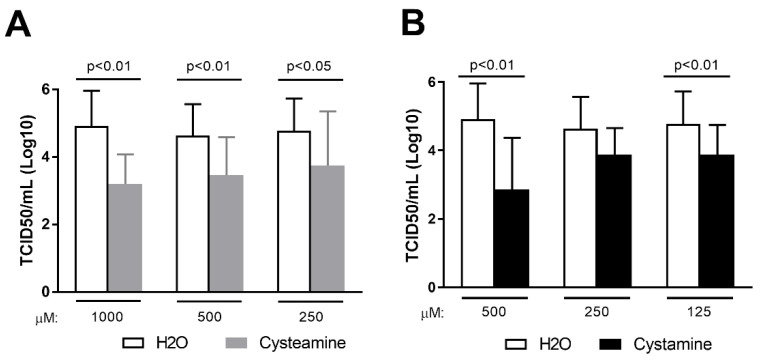
Cysteamine and cystamine decreased viral production in Calu-3 cells. (**A**,**B**) Calu-3 cells (0.375 × 10^6^ cells/well in 24-well plate) were treated with different doses of cysteamine (panel **A**) or cystamine (panel **B**), as indicated, or H_2_O as control 1 h before SARS-CoV-2 infection (MOI = 0.1). Absorption of the virus was allowed for 1 h at 37 °C in the presence of different treatments. The unabsorbed virus was removed and replaced by fresh medium with cysteamine, cystamine, or H_2_O. Cells were then treated every 24 h and incubated at 37 °C with 5% CO_2_ for 48 h when culture medium was collected. Virus yield was measured by back-titration of supernatants as described in the methods. Virus titers are expressed as 50% tissue-culture effective dose (TCID_50_/mL) values according to the Reed–Muench method. Statistically significant differences between compound- and H_2_O-treated cells have been calculated using Wilcoxon matched-pairs rank tests.

We next tested whether cysteamine and cystamine exert their antiviral activity in the lung-derived epithelial Calu-3 cells, as these cells are relevant as a model for human infections. Since SARS-CoV-2 did not induce CPE on Calu-3 cells, we evaluated viral production measuring the infectious virus yield (TCID50). Cells were pre-treated for 1 h with different doses of cysteamine, cystamine or H_2_O as control before SARS-CoV-2 infection (MOI = 0.1). Cells were then cultured in the presence of the drugs that were added 1 h and 24 h after infection until the end of the experiments (48 h.p.i.), when the culture medium was collected and viral titers measured. As shown in Figure 4, cysteamine and cystamine significantly reduced viral production in Calu-3 cell cultures, thus suggesting that these compounds exert a potent direct antiviral effect also in human cells, which are similar to the natural target of SARS-CoV-2.

### 3.5. Cysteamine and Cystamine Decrease the SARS-CoV-2-Specific Response in COVID-19 Patients

**Figure 5:**
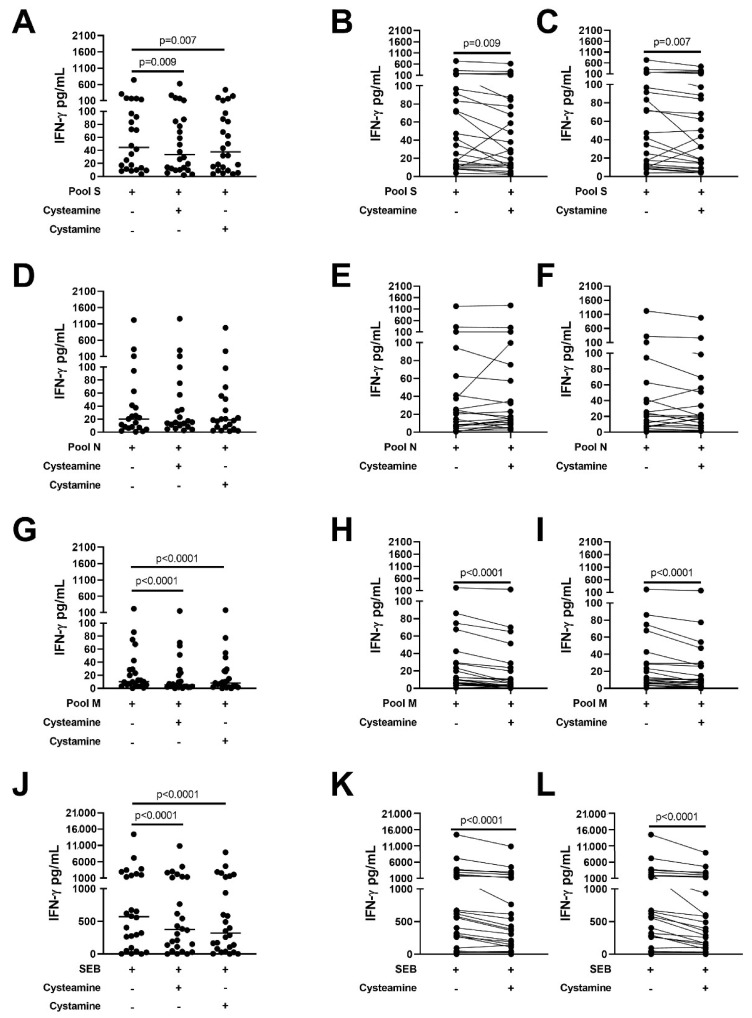
Cysteamine and cystamine decrease the SARS-CoV-2 specific response in COVID-19 patients. Analysis of cysteamine and cystamine effects on the in vitro IFN-γ response to SARS-CoV-2 peptide antigens evaluated in cells from the whole blood of COVID-19 patients. Cysteamine 400 µM and cystamine 200 µM significantly decreased IFN-γ levels in response to pool S (**A**–**C**), pool M (**G**–**I**), and SEB (**J**–**L**) compared to the untreated control; a decreasing trend induced by both cysteamine and cystamine was observed also for the IFN-γ response to pool N (**D**–**F**). The analysis was performed considering only the IFN-γ values >0 pg/mL. Left panels show the cumulative effects of cysteamine and cystamine on the IFN-γ response to the different stimuli. Right panels show effects of the two compounds on the IFN-γ response to the different stimuli in each evaluated subject. Horizontal lines represent medians. Wilcoxon test was applied. IFN-γ was measured by ELISA. Number of subjects analyzed: 24 patients (**A**–**C**), 21 patients (**D**–**F**), 23 patients (**G**–**I**), 26 patients (**J**–**L**). Footnotes: IFN: interferon; S: spike; N: nucleocapsid; M: membrane; SEB: staphylococcal enterotoxin B.

It is known that a dysregulated immune response is associated with COVID-19 disease severity [34], which is the rational for the use of drugs such as dexamethasone [35,36] or baricitinib [37,38,39] for COVID-19 treatment. Cysteamine has been shown to have anti-inflammatory properties [15,16,17]. Therefore, we evaluated whether cysteamine or cystamine had an immunomodulatory effect on the SARS-CoV-2-specific immune response. In particular, we evaluated the impact of these drugs on the in vitro IFN-γ-viral-specific response in the whole blood platform, as described [22,23,26]. Demographic and clinical characteristics of the 26 enrolled subjects are shown in Table 1. To better address the effect of these two compounds on the response to SARS-CoV-2 antigens (spike, membrane, and nucleocapsid), we analyzed the IFN-γ response considering only the IFN-γ values >0 pg/mL [22]. The analysis was, therefore, conducted on 24 patients for pool S (Figure 5A–C), 21 patients for pool N (Figure 5D–F), 23 patients for pool M (Figure 5G–I), and on 26 patients for SEB (Figure 5J–L).

Cysteamine or cystamine significantly decreased the IFN-γ levels in response to pool S (pool S median: 44.6 pg/mL, IQR: 12.0–128.6 pg/mL; pool S+cysteamine median: 33.6 pg/mL, IQR: 10.8–86.9; pool S+cystamine median: 37.7, IQR: 10.4–94.9) compared to the untreated control (*p* = 0.009 and *p* = 0.007, respectively) (Figure 5A). Figure 5B,C show the impact of cysteamine or cystamine on the IFN-γ production in stimulated whole blood from each single patient.

Similarly, cysteamine and cystamine, significantly decreased the IFN-γ response to pool M (pool M median: 10.4 pg/mL, IQR: 5.3–29.3; pool M+cysteamine median: 5.3 pg/mL, IQR: 2.1–23.8; pool M+cystamine median: 8.3 pg/mL, IQR: 2.1–26.5) compared to the untreated control (*p* < 0.0001 for both) (Figure 5G–I). Although not significant, a similar decreasing trend induced by both cysteamine and cystamine was observed also for the IFN-γ levels in response to pool N (pool N median: 20.3 pg/mL, IQR: 6.8–52.1; pool N+cysteamine median: 15.4 pg/mL, IQR: 9.3–66.3; pool N+cystamine median: 18.7 pg/mL, IQR: 6.6–53.3 pg/mL) (Figure 5D–F).

Interestingly, cysteamine and cystamine significantly reduced IFN-γ response to a polyclonal T cell activator, staphylococcal enterotoxin B, SEB (*p* < 0.0001) (SEB median: 569.7 pg/mL, IQR: 83.5–2037; SEB+cysteamine median: 374.7, IQR: 95.2–1374; SEB+cystamine median: 319.8, IQR: 69.3–142.1 *p* < 0.0001) (Figure 5J–L). Overall, these data indicated that cysteamine and cystamine decrease both the SARS-CoV-2-specific response and the SEB-mediated responses in blood from COVID-19 patients.

## 4. Discussion

In this study, we demonstrated that cysteamine and its disulfide product of oxidation cystamine display potent in vitro antiviral activity against SARS-CoV-2 and ex vivo immunomodulatory properties in blood cells from COVID-19 patients. These findings are particularly relevant since cysteamine is an approved drug routinely used as a gold standard for the treatment of cystinosis, a rare inherited autosomal recessive disease. Indeed, using different cell lines, we showed that treatment with cysteamine or cystamine exerted antiviral activity by impairing SARS-CoV-2 replication, as clearly highlighted by the presence of very few viral particles in the infected cells upon cysteamine treatment in the electron microscopy images. Moreover, drug treatment almost eliminated the typical cytopathic effects observed in untreated SARS-CoV-2-infected cells (i.e. rounded shape, grouped ribosomes, vacuolar degeneration consisting of accumulation of lipolysomes).

Interestingly, since the post-infection treatment has the same antiviral properties as the pre-infection treatment, the effects elicited by cysteamine or cystamine are likely due to the intracellular antiviral activity of the drugs and not to the reduced binding of the spike protein to the ACE2 receptor, as suggested for several thiol-based compounds [21]. Considering the antioxidant and immune-modulatory activities of cysteamine or cystamine [15,16,17], we evaluated the impact of these drugs on the in vitro SARS-CoV-2-specific response in blood from COVID-19 patients. The specific response was significantly reduced either to pool M or pool S, as well as upon SEB stimulation, indicating that cysteamine, in addition to the direct antiviral action, has also a potent immunomodulatory activity. This finding is particularly relevant considering that a dysregulated immune response is one of the hallmarks of COVID-19 characterized also by the so-called “cytokine storm” present in patients with severe disease. Indeed, it has been reported that aberrant pathogenic Th1 cells with co-expressing IFN-γ and GM-CSF are present in patients with severe disease compared to those with milder disease, suggesting that pathogenic Th1 cells may play a critical role in hyper-inflammatory responses during Covid-19 pathogenesis [40]. The role of type II IFN is currently poorly understood; it may be proviral in some sites by inducing expression of the viral receptor and may also drive inflammation. Indeed, treatment with neutralizing antibodies against TNF-α and IFN-γ protected mice from mortality during SARS-CoV-2 infection [41]. Furthermore, the percentage of IFN-γ-producing CD4^+^ T cells has been found increased in extremely severe patients, as well as the CD8^+^ T cells, thus indicating that the hyperfunction of these T cells could be associated with the pathogenesis of extremely severe SARS-CoV-2 infection [42] or merely with a specific response [43]. Current routine therapies are crucial for reducing the cytokine storm and are widely used depending on the disease stage of the patients [37,38].

Since treatment with cysteamine and cystamine may influence many intracellular and extracellular processes, it is difficult to draw definitive conclusions on the mechanism(s) responsible for the observed results. In fact, their free thiol group is highly reactive and can react with free thiol or the disulfide bounds of proteins, possibly interfering with their function such as, for instance, the cysteine protease caspase-3 or the tyrosinase dopa-oxidase [44,45]. Cysteamine promotes the transport of cysteine into cells promoting the glutathione synthesis, a potent intracellular antioxidant. It is known that the redox cellular state plays a key role in the regulation of many essential signaling pathways, including cell death and proliferation, and modulates the expression of several redox-sensitive genes.

Cysteamine and cystamine have been used as transglutaminase inhibitors. In particular, cysteamine inhibits transglutaminase by acting as a competitive inhibitor for the transamidation reactions catalyzed by this enzyme. Factor XIII (FXIII) is a plasma transglutaminase that crosslinks fibrin monomers and alpha(2)-plasmin inhibitor during clot formation; deficiency of FXIII worsens clot stability and increases bleeding. Interestingly, it has been shown that the transamidating activity of FXIIIa required for the proper clot stabilization is inhibited by cysteamine [46], thus the multifunctional effects of these drugs could also be beneficial in preventing the thrombosis associated with SARS-CoV-2 infection. In fact, a key aspect of the pathophysiology of COVID-19 is related to the hypercoagulable and hypofibrinolytic state observed in patients affected by severe disease, supported by the evidence of elevated D-dimer that represents the sign of excessive activation of coagulation. Many clinical data indicate a general beneficial effect of heparin for hospitalized COVID-19 patients, leading to a reduced mortality in subjects with markedly elevated D-dimer or high fibrinogen levels. Hence, the beneficial effects of cysteamine and cystamine during SARS-CoV-2 infection may exceed those observed in this study.

Although some scientific findings suggest that autophagy plays an important role in viral replication and pathogenesis, its role during SARS-CoV-2 infection remains to be completely defined. In keeping with this notion, we showed that cysteamine impairs the bacterium replication in Mtb-infected macrophages by inhibiting autophagy in vitro [14]. Thus, it would be important to explore in the future whether the cysteamine effects on SARS-CoV-2 are related to the modulation of cell autophagy, and how these events affect the host cell antiviral properties.

In conclusion, our findings indicate that cysteamine and cystamine modulate both viral replication and SARS-CoV-2-mediated immune response in vitro. These results are interesting to be further confirmed and expanded to animal models and humans as a new drug for COVID-19 treatment. Considering that cysteamine is a drug approved by the FDA and EMA, it would be important to conduct clinical trials to verify the therapeutic effect of these small molecules in COVID-19 patients. In particular, specifically designed clinical trials should validate the safety and the efficacy of cysteamine in clinical practice when used alone or in combination with other approved drugs.

## Figures and Tables

**Table 1:** Demographical and clinical characteristics of the COVID-19 enrolled patients.

	COVID-19
N (%)	26
Age median (IQR)	60 (50–69)
Male N (%)	18 (69.2)
Origin N (%)	
West Europe	22 (84.6)
Asia	4 (15.4)
Swab positive results N (%)	26 (100.0)
Severity N (%)	
mild	1 (3.8)
moderate	10 (38.5)
severe	15 (57.7)
critical	0 (0)
Steroid therapy	
Yes	14
No	12
Days of steroid therapy median (IQR)	4 (3–6)

COVID-19: COronaVIrus Disease 2019; N: Number.

## Data Availability

The raw data generated and/or analyzed within the present study are available in our institutional repository (rawdata.inmi.it), subject to registration. The data can be found by selecting the article of interest from a list of articles ordered by year of publication. No charge for granting access to data is required. In the event of a malfunction of the application, the request can be sent directly by e-mail to the Library (biblioteca@inmi.it).

## Supplementary Materials

The following are available online at https://www.mdpi.com/article/10.3390/cells11010052/s1; Supplementary Figure S1, Cysteamine and cystamine decreased viral RNA production in Vero E6 cells. Supplementary Figure S2, Cysteamine treatment reduce SARS-CoV-2 production and decrease the number of infected Vero E6 cells. Supplementary Figure S3, Electron microscopy images of Uninfected VERO E6 cells. Supplementary Figure S4, TG2 is not involved in SARS-CoV-2 life cycle.
