# Supplementary material for: Cysteamine with In Vitro Antiviral Activity and Immunomodulatory Effects Has the Potential to Be a Repurposing Drug Candidate for COVID-19 Therapy
Source: Cells. 2021 Dec 24;11(1):52. doi: 10.3390/cells11010052 (PMC8750154; doi:10.3390/cells11010052)
Supplement: Supplementary file 1 [file cells-11-00052-s001.zip › cells-1493006 supplementary materials proofreading back.pdf]

Supplementary Materials

Supplementary Figure 1

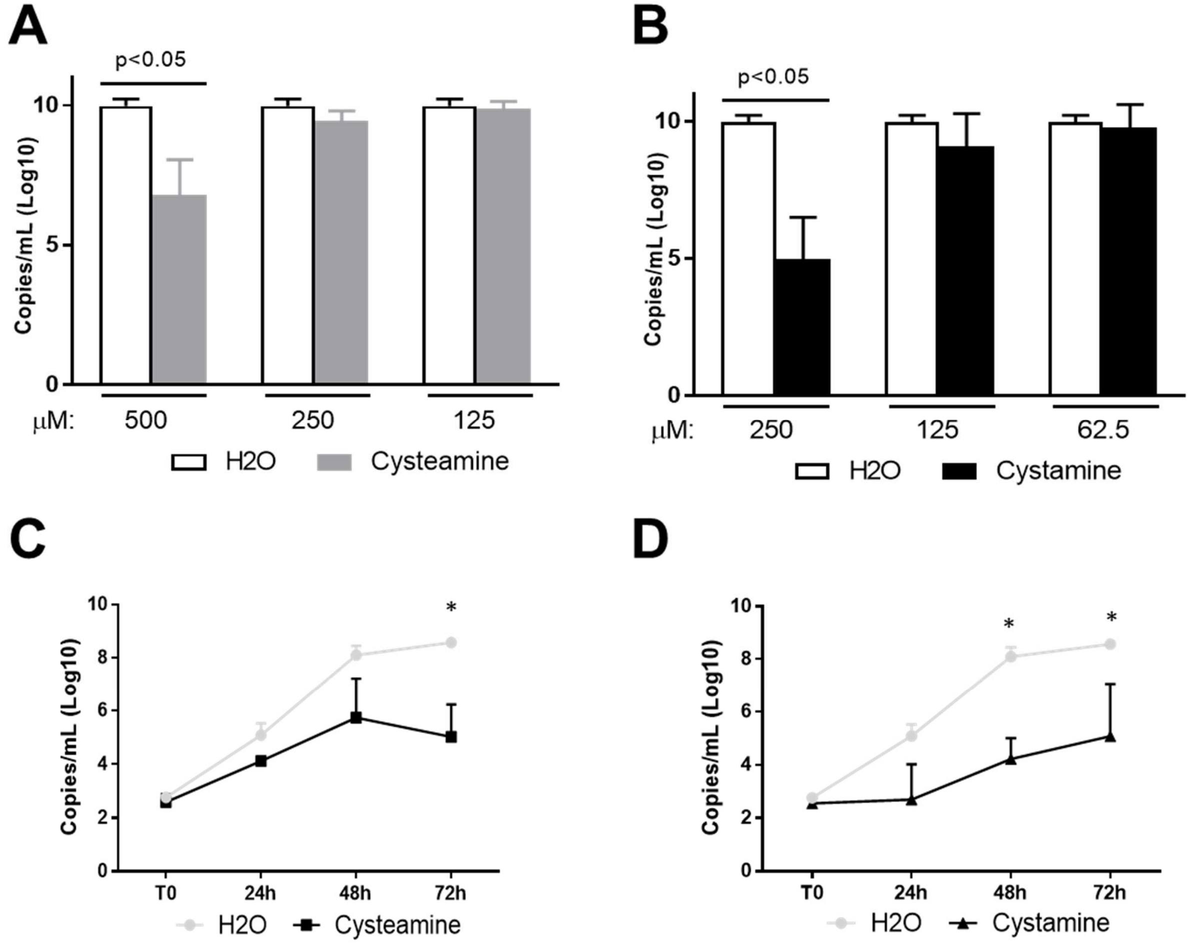

Figure S1. Cysteamine and cystamine decreased viral RNA production in Vero E6 cells. (A-B) Vero E6 cells were treated with different doses of cysteamine (panel A) or cystamine (panel B) as indicated or the corresponding volume of H_2O as control 1h before SARS-CoV-2 infection. Cells were then treated every 24h and incubated at 37°C with 5% CO_2 for 72h as in figure1. Culture media of SARS-CoV-2-infected Vero E6 cells, treated as indicated, were collected at 72h.p.i. Virus RNA was measured by qRT-PCR as described in the methods. (C-D) Vero E6 cells were treated with cysteamine (500μM panel C) or cystamine (250μM panel D) or the corresponding volume of H_2O as control 1h before SARS-CoV-2 infection. Cells were then treated every 24h and incubated at 37°C with 5% CO_2 for the indicated times when culture supernatants were collected and viral RNA measured by qRT-PCR. The results are the means±SD of 3 independent experiments. * $p<0.05$.

Supplementary Figure 2

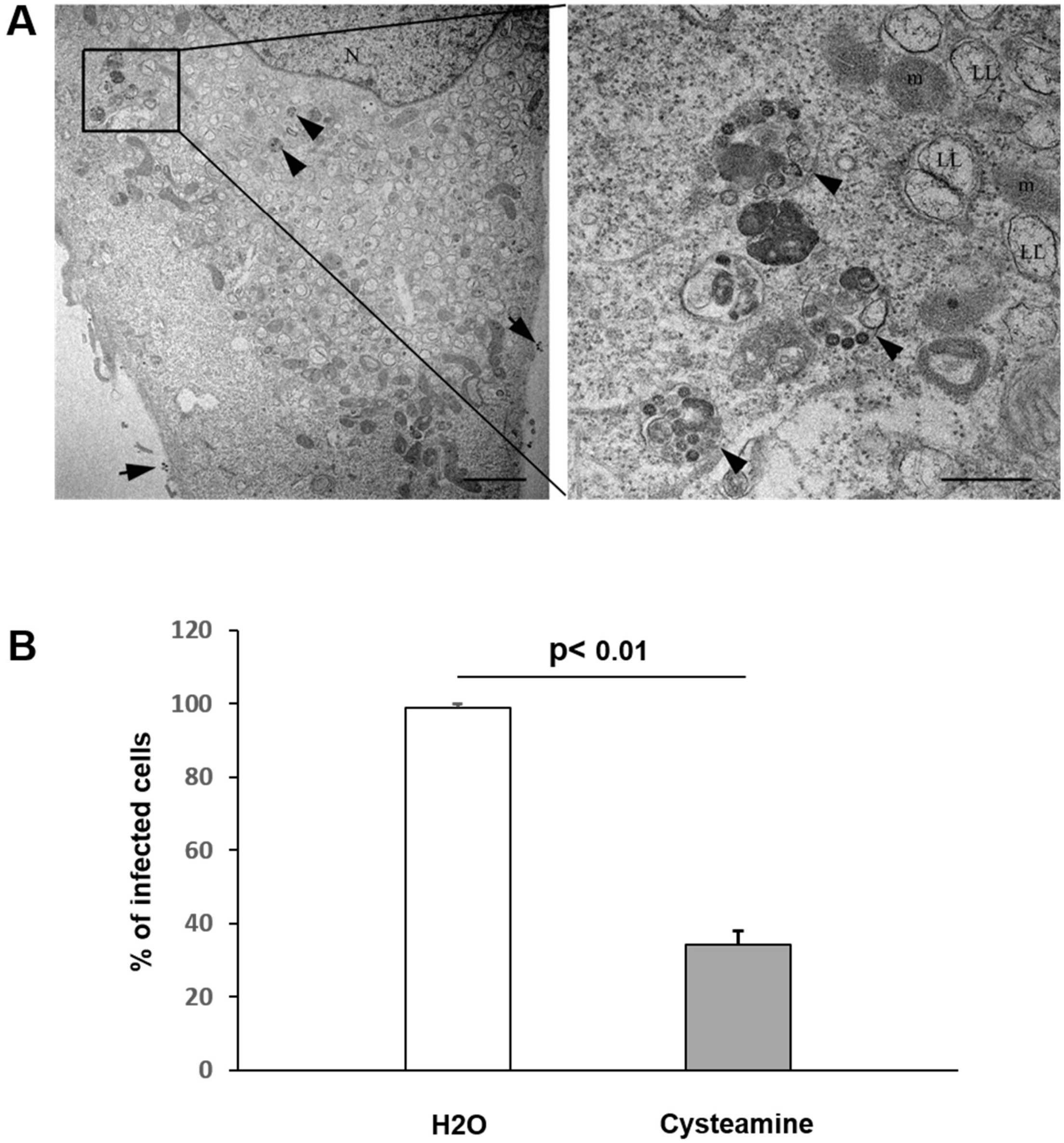

Figure S2. Cysteamine treatment reduced SARS-CoV-2 production and decreased the number of infected Vero E6 cells. (A) (Left Panel) Transmission electron micrograph showing one cysteamine-treated cell with rare particles at the cell surface (arrows) and few intracytoplasmic vacuoles containing virions (arrowheads). (Right Panel) Higher magnification of the boxed area, displaying SARS-CoV-2 particles located in vacuolar structures (arrowheads) and lipolysosomes (LL). (B) The graph shows the percentage of infected cells, 48 h post infection, untreated or treated with cysteamine (500 μM). Data are mean ± SD from three independent experiments. Statistically significant difference is showed ($p < 0.01$). using unpaired, two-tailed Student's t-test. N, nucleus; LL, lipolysosomes. Scale bars: Left Panel, 2 μm; Right Panel = 500nm.

Supplementary Figure 3

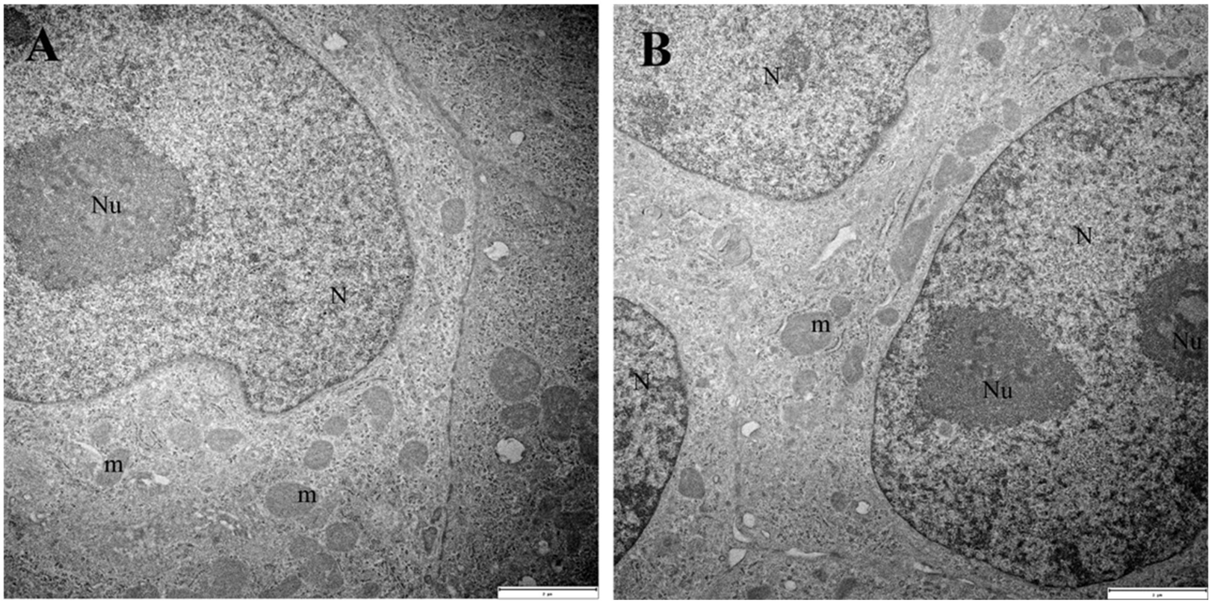

Figure S3. Electron microscopy images of not infected Vero E6 cells. (**A**) Ultrastructural appearance of not infected Vero E6 cells cultured for 48h. (B) Vero E6 cells cultured for 48h and treated with 500 μm cysteamine. The cells showed their normal morphology, no signs of intracellular alterations after the cysteamine treatment are pointed out. N, nucleus; Nu, nucleolus; m, mitochondria. Scale bars: A,B=2μm.

Supplementary Figure 4

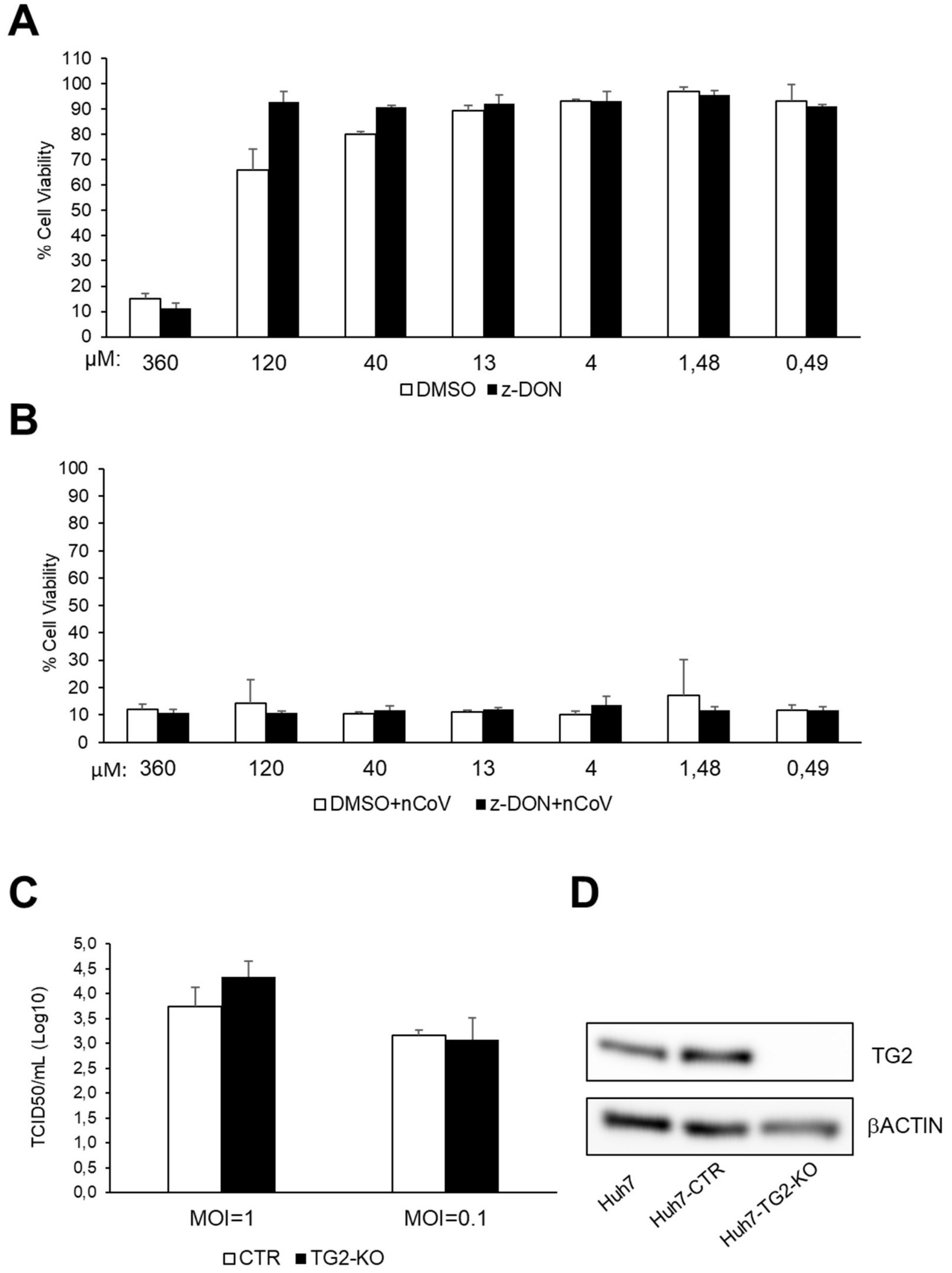

Figure S4. TG2 is not involved in SARS-CoV-2 life cycle. (A-B) Vero E6 cells were treated with different doses of Z-DON (from 360 to 0.49µM; 1:3 serial dilutions; black columns) or DMSO as control (from 0.9 to 0.001% v/v; 1:3 serial dilutions; white columns) 1h before SARS-CoV-2 infection (MOI=0.001). Absorption of the virus was allowed for 1h at 37°C in presence of the different treatments. The unabsorbed virus was removed and replaced by fresh medium with Z-DON or DMSO as above. Cells were then treated every 24h and incubated at 37°C with 5% CO_2 for 72h when the survival of not infected (A) or infected (B) cells was measured by crystal violet staining assay. The results were evaluated setting the not infected cells as 100% and the remaining values represented as a relative value. Experiments were performed in triplicate and data are expressed as mean ± S.D. (n = 2). (C) Culture media of SARS-CoV-2-infected Huh7-CTR (white columns) or Huh7-TG2-KO (black columns) cells, were collected at 48h.p.i. Virus yield was measured by back-titration of supernatants as described in the methods. Virus titers are expressed as 50% tissue-culture effective dose ($TCID_{50}$/mL) values according to the Reed-Muench method. Experiments were performed in triplicate and data are expressed as mean ± S.D. (n = 2). (D) Immunoblotting analysis of TG2 in Huh7 parental cells (Huh7) and in which TG2 gene has been deleted (Huh7-TG2-KO), with its negative control (Huh7-CTR), by CRISPR/Cas9 technology. Beta-actin levels were used as loading control.
